# Effects of digital game-based learning as a tool for laparoscopy training in surgical nursing

**DOI:** 10.1371/journal.pone.0336400

**Published:** 2026-02-03

**Authors:** Fatemeh Akbari HajiAbad, Fatemeh Keshmiri, Fatemeh Jabinian

**Affiliations:** 1 Department of Operating Room, School of Paramedical, Shahid Sadoughi University of Medical Sciences, Yazd, Iran; 2 Student Research Committee, Shahid Sadoughi University of Medical Sciences, Yazd, Iran; 3 Medical Education Department, Education Development Center, Shahid Sadoughi University of Medical Sciences, Yazd, Iran; Medical Park Minatomirai, JAPAN

## Abstract

**Background:**

The present study aimed to (1) develop a digital game focusing on the roles of surgical nurses (scrub and circulator nurses) in laparoscopic surgeries, (2) conduct a randomized controlled trial (RCT) comparing this digital game-based learning (GBL) approach with a conventional teaching method, and (3) assess students’ reasoning and user satisfaction.

**Method:**

This randomized controlled trial (RCT) was conducted at Shahid Sadoughi University of Medical Sciences. The study comprised three phases: development of the digital game titled ‘Co-Surgeon,’ implementation of the educational interventions, and evaluation of outcomes. The digital game aimed to enhance students’ understanding of laparoscopic surgeries and the specific roles of surgical nurses (circulator and scrub), focusing on tasks such as task recognition, laparoscopic instrument identification and utilization, procedural steps, and the development of clinical reasoning for selecting appropriate tools during common laparoscopic procedures. Fifty-seven surgical nursing students were randomly assigned to either an intervention group, which utilized digital game-based learning, or a control group, which received conventional lecture-based education. The digital GBL application included 40 distinct laparoscopic tools and equipment organized into 8 categories, 8 puzzles related to surgical nurse roles in abdominal laparoscopic surgeries, 15 Mayo stand setups, and 60 instrument identification puzzles. User satisfaction was measured via the Questionnaire for User Interface Satisfaction (QUIS), and students’ reasoning was assessed through the Key Feature (KF) examination. Data were summarized using descriptive statistics (mean, standard deviation, and percentage). Pearson correlation coefficient, independent t-test, and paired t-test were used for data analysis.

**Results:**

The mean (SD) scores of students’ reasoning in the intervention group (19.51 ± 3.36) were significantly higher than those in the control group (13.92 ± 4.15). (p < 0.001), with a large educational effect size reported (Partial eta squared = 0.35). Student perception scores indicated a good level of satisfaction (184.85 ± 6.79).

**Conclusion:**

The diverse and complex responsibilities of surgical nurses make it essential to enhance the learning outcomes for surgical nursing students. Implementing digital game-based learning can positively impact these outcomes. Therefore, it is recommended to incorporate digital games as a supplement in clinical education and workplace-based training.

## Introduction

Complex technology in minimally invasive surgeries, such as laparoscopy, offers significant benefits for surgical outcomes. However, it also increases the cognitive load on surgical team members. Elevated cognitive load in stressful situations can lead to increased errors [1].

Game-based learning (GBL), commonly known in medical education as “serious games,” “educational games,” and “gamification,” is utilized across both basic science and clinical courses. [2]. GBL combines learning objectives with engaging challenges, incorporating competitive elements, feedback mechanisms, and an enjoyable experience to engage learners [1,3]. As a fundamental educational method for laparoscopic training, GBL offers learners valuable opportunities to prepare for laparoscopic surgery by undertaking various roles [1,3,4]. Effective GBL requires learners to develop skills such as rapid information processing (processing speed), working memory retention, cognitive flexibility (cognitive control), and problem-solving (reasoning). The inclusion of visual elements enhances learners’ awareness, deepens conceptual understanding, and improves reasoning and learning outcomes [5,6]. Michalewicz recommends incorporating puzzles within GBL to promote creative decision-making, build confidence in problem-solving, and stimulate memory formation for cohesive cognitive schemas [7]. Well-designed GBL increases learner participation, motivation, enthusiasm, and interest, creating an optimal memory-making environment [8]. Enhancement of learning, problem-solving skills, reasoning, and clinical decision-making are consistently reported benefits of GBL [2,8,9]. This evidence highlights the effectiveness of GBL as a pedagogical strategy in medical education for developing essential cognitive and clinical skills.

Malicki and colleagues investigated the use of interactive digital learning in nursing education, focusing on games, gamification, and scenario-based learning. Their findings confirmed that games have a positive effect on learner participation and satisfaction. However, they noted that further research is needed to assess the impact of game-based learning (GBL) on learner engagement, cognitive function, and knowledge retention in nursing education [10]. Similarly, Graafland et al. recommended further studies to assess GBL’s impact on both technical and non-technical skills [1]. The present study aimed to [1] develop a digital game focused on the roles of surgical nurses (scrub and circulator nurses) in laparoscopic surgeries, [2] implement a randomized controlled trial (RCT) comparing this digital game-based learning with conventional teaching methods, and [3] assess surgical students’ reasoning and user satisfaction.

## Materials and methods

### Study design, setting, and participants

This randomized controlled trial (RCT) was conducted at Shahid Sadoughi University of Medical Sciences (SSU) from 1 November 2022–25 October 2023. This exploratory RCT is part of a larger study designed primarily to investigate the feasibility and educational impact of game-based learning in three stages: game development, implementation, and assessment, focused on surgical nursing students.

## Participants

### Development of digital GBL

Fifteen experts contributed to the development of the GBL conceptual framework, including defining learning objectives, learning activities, and game dynamics, as well as conducting face and content validity assessments. The panel consisted of ten surgical nurses (66.7%) and five laparoscopic surgeons (33.3%). The mean age of experts was 48 years (SD = 9), with an average work experience of 18 years (SD = 13).

The game development team comprised five members specializing in educational design and clinical practice. The team consisted of five members: an expert in health professions education (Ph.D.), a surgical nursing instructor (MSc), a surgical nurse (MSc), a general surgery specialist (MD, Professor of Surgery), and an information technology specialist (MSc). The average age of the team was 45 years (SD = 12), and they had an average of 16 years of work experience (SD = 10).

### Implementation of the intervention

Participants were third- and fourth-year surgical nursing students enrolled at Shahid Sadoughi Hospital, affiliated with SSU. Inclusion criteria required completion of the surgical instrument management course and informed consent to participate. Students with prior laparoscopic surgery experience were excluded. Eligible students were randomly assigned by lottery to either the intervention or control group. Written informed consent was obtained from all participants.

**Fig 1:**
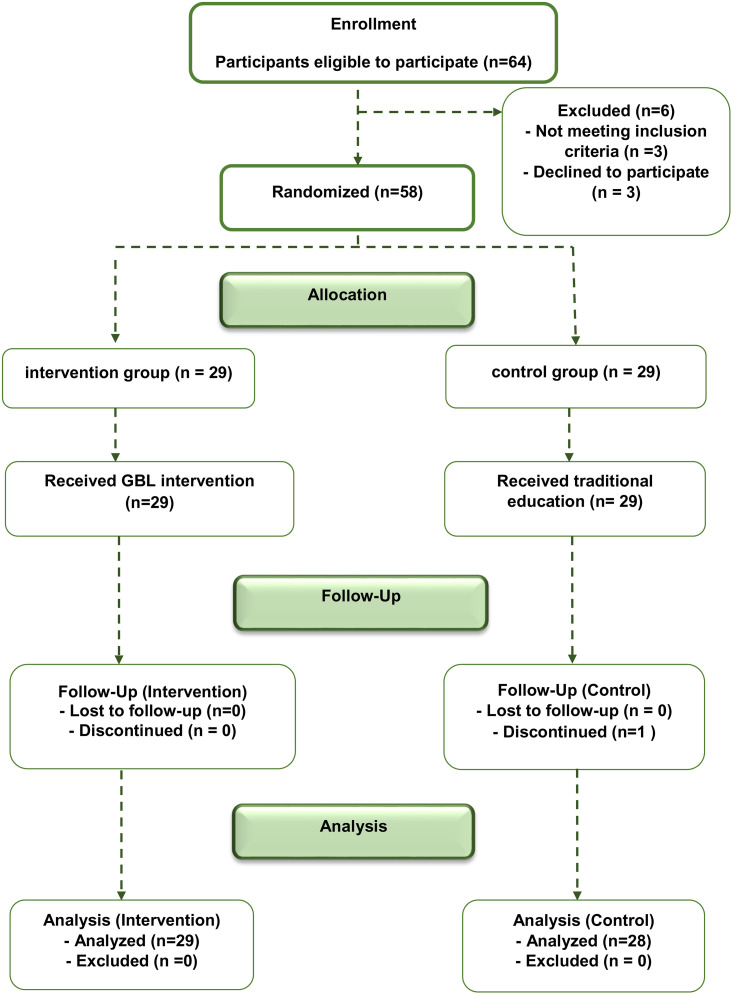
Flow chart of the study steps.

Sample size calculation, based on a Type I error rate of 5%, 80% statistical power, and an effect size of 0.08 derived from a pilot study, indicated 26 students per group were needed. To accommodate an anticipated 10% dropout, the total sample size was increased to 28 per group. A total of 57 students participated in the intervention phase (Fig 1).

**Facilitator/instructor:** Two experts in surgical nursing conducted the educational sessions for both groups. They included one surgical nursing instructor (MSc) and one surgical nurse (MSc). Their mean age was 34 years (SD = 2), with an average work experience of 8 years (SD = 3).

## Study phase

### Digital game-based learning design

**Conceptual Framework:** This GBL intervention aimed to enhance the learning of surgical nursing students in laparoscopic surgeries. It was designed using a concept mapping exercise as a knowledge-oriented instructional strategy to teach clinical reasoning combined with puzzle-based learning.

Concept mapping was employed as a core instructional strategy to teach clinical reasoning. The Clinical Reasoning Mapping Exercise (CReSME) is a highly structured concept map providing learners with a graphical representation of knowledge structures, serving as a scaffold for developing early cognitive scripts. It requires learners to connect information precisely and meaningfully, facilitating comparison and analysis of key features of various activities related to a concept across multiple domains [11].

Puzzle-based learning was introduced to improve critical thinking, problem-solving, and decision-making skills. Its goal is to build a foundation for learners to become effective problem solvers in real-world contexts by encouraging them to frame and solve problems [7]. Successful problem solvers utilize three skill categories: managing uncertain and dynamic situations; integrating domain-specific knowledge and methods; and applying critical thinking with general problem-solving strategies [7]. Puzzle-based learning supports criteria such as generality (preparing learners to solve future real problems), simplicity (enabling rapid memory encoding of puzzles and solutions), entertainment (motivating learners through engagement), and Eureka moments (structured activities with immediate feedback and rewards) [6,7].

To design the game, a literature review was conducted on digital games relevant to laparoscopic surgery and nursing education [1,10,12–15]. Expert opinions were sought regarding educational requirements, common challenges, and critical errors faced by surgical nurses in laparoscopic surgery. Based on this combined input, an expert panel including members of the design team established the educational objectives. Subsequently, specific educational objectives and correlated learning activities were formulated within this conceptual framework, ensuring alignment with established expert viewpoints.

**Educational objectives:** The primary educational objective of the game was to enhance learners’ understanding of laparoscopic surgeries and the roles of surgical nurses, specifically the circulator (the non-sterile team member) and the scrub nurse (the sterile team member). This included tasks such as recognizing, identifying, and utilizing laparoscopic instruments, understanding the steps of surgical procedures, and developing reasoning skills for selecting the appropriate tools for common laparoscopic surgeries. Further, Students were expected to set up a Mayo stand for each surgery. A summary of the detailed educational objectives is provided in Table 1.

**Table 1:** Educational objectives of the GBL.

The student should be able to: Recognize the tasks and roles of surgical nurses during surgical procedures Identify various types of laparoscopic instruments and their classifications Differentiate the applications of each laparoscopic tool and piece of equipment Select the appropriate instrument among similar laparoscopic tools Properly arrange the surgical instrument trolley as a scrub nurse Identify the correct use of instruments at each step of the surgical procedure

**Content of Digital GBL:** Common laparoscopic procedures, namely cholecystectomy, appendectomy, inguinal herniorrhaphy, and sleeve gastrectomy, were selected as the foundational content for the game by experts.

**Digital GBL Sections**: The GBL comprises two main sections.

1) Puzzle of Surgical Nursing Tasks

**Fig 2:**
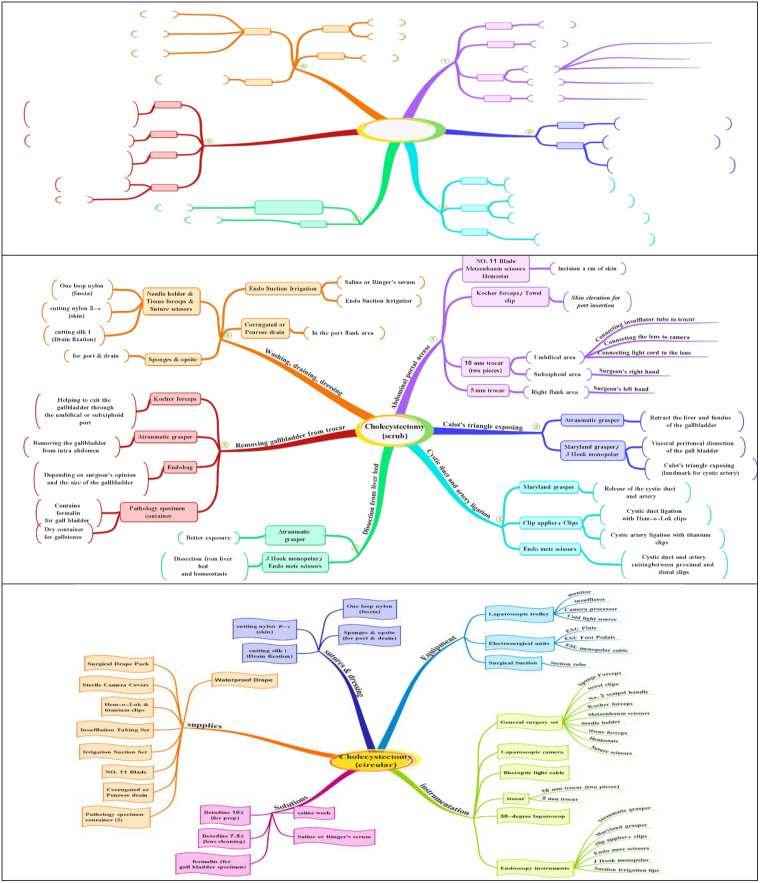
Puzzle of Surgical Nursing Tasks in digital GBL of Co-surgeon.

This section focuses on the dual roles of a surgical nurse—scrub and circulator—in laparoscopic surgeries. (Fig 2).

**Fig 3:**
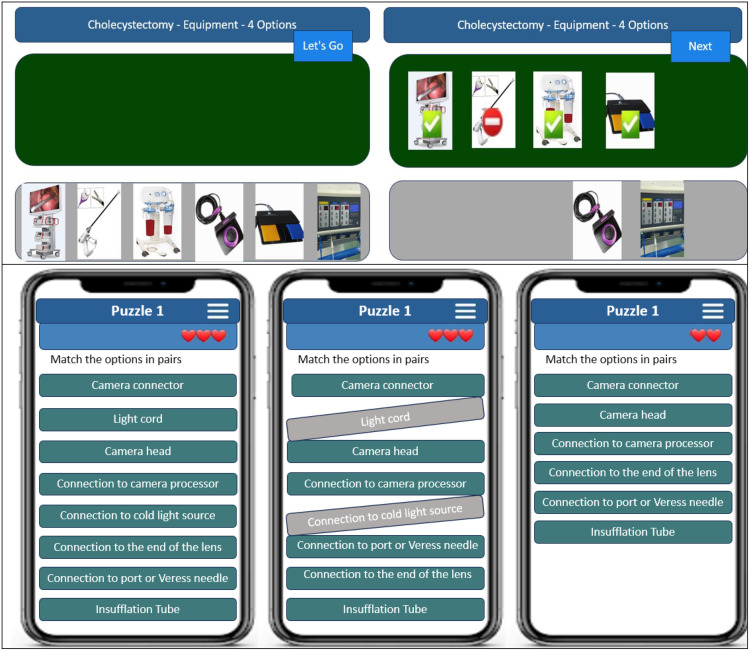
Puzzle of Surgical Instrumentation in digital GBL of Co-surgeon.

- In the puzzles for the scrub role, learners complete tasks by ordering surgical steps, identifying instruments used in each step, and applying each instrument according to the scenario. This section emphasizes a detailed understanding and recognition of tool usage at each surgical stage.
- In the puzzles for the circulator role, learners select appropriate tools—including equipment, instrumentation, solutions, supplies, sutures, and dressings—to prepare for surgery based on the scenario.

2) Puzzle of Surgical Instrumentation

- **Mayo stand setup**: Learners select appropriate tools and equipment for each specific laparoscopic surgery setup.
- **Instrument identification**: Learners recognize instruments by their features and shape, organizing them according to the steps of the surgery. (Fig 3).

Game scenarios for laparoscopic surgeries were developed using both static and animated images to enhance their visual appeal. Each activity provided prompt and constructive feedback to the learners. The game scenarios were evaluated for validity by 15 surgical experts in individual meetings. Their feedback and suggestions were documented and analyzed by the expert panel of the game design team, resulting in necessary revisions. Ultimately, the experts confirmed the content validity of both the game scenarios and the puzzle formats.

### Digital game development

The first version of the digital GBL application, entitled ‘Co-Surgeon,’ was developed in five months (S1 Appendix). The ‘Co-Surgeon’ is a digital game designed for smartphones operating on Android and iOS platforms. The design team reviewed the initial version and offered suggestions for improvement. Some suggestions included designing maps for single-page viewability, enabling zoom functionality for concept maps, and using visual aids such as images and animated GIFs to demonstrate laparoscopic surgical tools in key game sections. Additional feedback included adjusting the Mayo stand setup activity to reflect real-world proportions and adding appropriate colors, along with modifications based on the nurse’s role within the game context. The second version underwent a three-month review by the design team and was subsequently approved. Afterwards, the face and content validity of the game were confirmed by 15 experts involved in the initial review.

This game includes 40 specific laparoscopic tools and equipment organized into 8 categories (grasper, retractor, cutter, dissector, scope, suture and stapler, laparoscopy equipment, accessory instruments). Moreover, the game contains 8 puzzles reflecting surgical nurse roles (four puzzles for scrub nurses and four for circulating nurses) in abdominal laparoscopic surgeries, 15 Mayo stand setup activities, and 60 instrument identification puzzles. A total of approximately 150 static images and 60 animated GIFs were utilized. The game also features a forum and instructional section. Activity timing was set for each step, with players earning coins for correct answers and losing coins for incorrect choices. Feedback included images and explanations across all activities.

### Education Implementation in the intervention group

Students in the intervention group attended a debriefing session to familiarize themselves with the digital GBL application and its components. They then played the game for three weeks, dedicating at least 6 hours. Accumulated points in the game verified participation. During this period, learners could communicate with the facilitator for questions or feedback. The facilitator monitored each student’s involvement, progress, and in-game performance (e.g., coins earned, puzzles completed) via the game’s admin panel. Individualized weekly feedback was emailed to each student, summarizing engagement compared to group averages and providing constructive feedback on puzzle performance, highlighting strengths and areas needing improvement.

### Education Implementation in the control group

For the control group, three educational sessions were conducted over three weeks using traditional lectures and group activities. The content of these sessions was identical to that provided to the intervention group. These sessions included the display of images related to instruments and surgical procedures, followed by discussions among learners about the applications of those instruments. To avoid any overlap between the groups, the control sessions were held before the intervention group sessions.

### Assessment of learner-related outcome

Learners in both groups undertook a supervised examination one week before and two weeks after the training.

### Study measures

Students’ reasoning was evaluated using a Key Feature (KF) examination, developed by two surgical nursing instructors based on guidelines by Nayer and colleagues [16]. The KF exam included twelve closed-ended questions, each with a scenario, a lead-in question, and 12 options. Learners selected three options per question, with scores ranging from 0 to 32.

The validity of the questions was assessed through qualitative and quantitative content validity indices (CVR and CVI) by 15 surgical nurses and laparoscopic surgeons. All questions met face and content validity criteria (CVR > 0.49, CVI > 0.79). [17,18] Internal consistency was confirmed with Cronbach’s alpha = 0.80 in a pilot test with 22 nurses.

Learners’ perception of the digital game was measured using the Questionnaire for User Interface Satisfaction (QUIS), comprising 27 items across five domains: feedback during software use (6 items), display interface (4 items), software terminology and information (6 items), software learnability (6 items), and overall software capabilities (5 items). Items were rated from 0 (lowest satisfaction) to 9 (highest), classified as weak (0–2), moderate (34–5), good (67–8), and excellent [9,19]. The questionnaire was previously validated in this context by Mahdizadeh et al. [20].

### Data analysis

The data were summarized using descriptive statistics (mean, standard deviation, and percentage).

Pearson correlation and independent t-tests were used to compare scores between groups. Paired t-tests evaluated score changes before and after the intervention. Effect sizes were reported using Partial Eta Squared (η²), with thresholds of approximately 0.01 (small), 0.06 (medium), and 0.14 (large). [21]. The level of significance was set at 0.05.

### Ethical consideration and consent

This study, part of a larger project on laparoscopy education among surgical nursing students [22], was approved by Shahid Sadoughi University of Medical Sciences Research Ethics Committee (ID: IR.SSU.REC.1400.199). Written informed consent was obtained from all participants. To comply with ethical protocols, the control group received a debriefing and access to the ‘Co-Surgeon’ application after the post-test and before final exams, ensuring an equitable opportunity to benefit from the educational technology. Technical support was provided for smooth onboarding. During manuscript preparation, an AI-based language model was utilized for proofreading and structural refinement while maintaining academic rigor.

The *GBL* application for this study, including the ‘co-surgeon’ application and Key Features Questions, was designed and developed by the authors, who retain all intellectual property rights. Ethical approval for the development and use of this application was obtained (IR.SSU.REC.1400.199, provided in the appendix). The GBL application and questionnaires used in this research—including the ‘co-surgeon’ module and Key Features Questions—are original to this study, except for the Questionnaire for User Interface Satisfaction (QUIS), which was adapted from Mahdizadeh’s study [20]. No other copyrighted or proprietary tools requiring external permission were used in this study

## Results

Fifty-seven surgical nursing students participated: 29 (52%) in the intervention group and 28 (48%) in the control group. The mean (SD) age was 21.6 (1.3) years. Participant demographics are shown in Table 2.

**Table 2:** Demographic information of the participants.

Demographic information	Categories	Group				P-value
		Intervention N = 29		Control N = 28		
		N	%	N	%	
Gender	Female	23	73.9	17	60.7	0.125
	Male	6	20.7	11	39.3	
Semester	5	11	37.9	8	28.6	0.702
	6	11	37.9	11	39.3	
	7	7	24.1	9	32.1	
GPA scores	<16	3	10.3	4	14.3	0.845
	16-18	23	79.3	22	78.6	
	>18	3	10.3	2	7.1	

Reasoning scores in the intervention group were significantly higher than those in the control group (p < 0.001). Both groups showed significant pre- to post-intervention score improvements (intervention p < 0.001; control p = 0.022). The mean difference in scores was significantly greater in the intervention group, indicating a stronger effect of the GBL intervention compared to the conventional method. (p < 0.001).

No significant association was found between learning scores and students’ GPA in either group (p > 0.05) (Table 3).

**Table 3:** Learning scores of students in intervention and control groups.

Time of assessment	Intervention group	Control group	95% Confidence Interval of the Difference		P-value*	Before and after mean difference	P-value	95% Confidence Interval of the Difference		Partial eta squared
	Mean ± SD	Mean ± SD	Lower	Upper		Mean ± SD		Lower	Upper	
Pre-test	12.77 ± 4.08	11.87 ± 3.21	−2.85	1.04	0.353	2.05 ± 4.46	> 0.001	−7.14	−2.22	0.35
Post-test	19.51 ± 3.36	13.92 ± 4.15	−7.59	−3.58	>0.001	6.47 ± 4.79		−7.14	−2.23	

* The significance level was calculated by the independent t-test at a confidence level of 95%.

Learners’ perception scores (Table 4) indicated a good overall satisfaction, with the highest scores observed in the software learnability domain.

**Table 4:** The satisfaction scores of learners about the digital game.

Domain	Mean	SD
Software function	41.28	5.2
Display screen	27.48	9
Software terms and information	41.17	2.4
Software learnability	42.83	7.5
General features of the software	32.07	7.2
Total	184.85	6.79

## Discussion

In clinical education, GBL is introduced to develop learners’ knowledge and clinical reasoning [2,13]. The findings indicate that the GBL positively impacted the reasoning of surgical nursing students, with the educational effect of the intervention reported at a high level.

Surgical nurses play a crucial role in the operating room, requiring a comprehensive understanding of surgical tasks and ensuring proper preparation of instruments at each stage. Step-by-step learning facilitates students’ conceptual visualization of surgical procedures within clinical environments and supports the retrieval of categorized scripts in future similar situations [9].

This study employs puzzle-based GBL designed to teach laparoscopic surgical procedures and the specialized roles of surgical nurses, focusing not only on technical knowledge but also on developing reasoning and decision-making skills. The novel aspect of this research is its dual-role puzzle design targeting both scrub nurses (sterile team members) and circulator nurses (non-sterile team members). These puzzles require learners to understand task sequencing, instrument selection based on surgical scenarios, and correct Mayo stand setup for each laparoscopic surgery. Such a comprehensive and systematic educational design integrates practical skills with cognitive recognition of surgical instruments and procedural steps [6].

In this GBL, students were exposed to scenarios requiring active engagement to complete each stage of the puzzles step-by-step, following the principles of both scrub and circulator roles. Moreover, learners received feedback at every puzzle stage to reinforce learning. Koivisto et al. recognized that GBL enhances nurses’ reasoning and decision-making skills by engaging students with patient problems, encouraging active participation in problem-solving, utilizing prior knowledge, and promoting feedback and reflection [23]. Likewise, Akbari et al. reported that a Mindtools strategy in mobile-based learning, used as a complementary tool in clinical education, enhanced both knowledge and self-efficacy among surgical nursing students [22]. Khorammakan identified that GBL facilitates content mastery, promotes analogical and clinical reasoning, and improves learners’ problem-solving skills through strategy formulation, reorganization, and planning [6]. In alignment with our GBL, Graafland and colleagues applied GBL in laparoscopy to create challenging problem-solving and decision-making opportunities, with results showing positive effects on learners’ situational awareness—a critical cognitive skill [3].

Laparoscopic surgical procedures are complex, multi-step processes that require improving team members’ learning to enable quick and precise decision-making. One critical aspect of surgical nursing is a thorough understanding of surgical tools and mastery of their application at each stage of laparoscopic surgery. Given the large number of instruments and their similar appearances, precise recognition and correct application of instruments at each surgical step were emphasized as key educational objectives in this game. In the digital GBL, students were assigned to select suitable surgical tasks, suggest instruments for each stage of the procedure, and receive immediate feedback. One vital task for surgical nurses in the operating room, setting up a Mayo stand, was digitally simulated in the game. Learners faced scenarios where they had to choose the correct tools and arrange them according to surgical order and principles. They also set up Mayo stands for various surgeries and received feedback on their performance in puzzle activities. This aligns with puzzle-based learning, which presents, discusses, and clarifies problem-solving principles through engaging and illustrative puzzles. Michalewicz and colleagues stated that puzzle-based learning facilitates students’ experiential understanding of problem-solving principles and exposes them to essential educational concepts [7]. Likewise, Khorammakan’s findings align with ours, showing that digital gaming enhanced surgical technology students’ knowledge and cognitive function in Coronary Artery Bypass Graft surgery [6]. Deschênes and colleagues, in a review study, recognized that feedback, trial-and-error, iterative learning, and gamification enhance students’ reasoning and decision-making processes by facilitating cognitive script formation. Incorporating these elements into complementary educational tools, such as GBL, positively affects key cognitive functions like reasoning and decision-making [12].

Game-based learning is a valuable approach for achieving learning outcomes, particularly in the cognitive domain [2,14,24]. The current study’s results demonstrated that students in the intervention group who engaged in GBL achieved significantly higher reasoning scores than those in the control group. Active participation in solving puzzles related to various laparoscopic activities positively influenced these outcomes. In line with these findings, Ozdemir reported that integrating game-based learning into nursing curricula enhances students’ cognitive learning [14]. Coelho and colleagues highlighted puzzles as complementary learning tools, enabling students to engage with complex surgical processes and develop their own procedural steps without risking patient safety [25]. Cardozo et al. (2021) similarly found that puzzles within digital GBL positively impacted students’ learning [26]. Boeker’s study compared learning outcomes between an intervention group experiencing GBL and a control group receiving conventional script-based education using true/false questions. Their results indicated that the intervention group not only scored higher but also exhibited more positive attitudes towards learning compared to controls [27]. In the present study, reasoning was assessed using a Key Feature examination, which evaluates learners’ reasoning by presenting novel scenarios requiring cognitive problem-solving. This method is recommended for assessing clinical reasoning in health professions education systems [16]. Our results are consistent with Boeker’s findings, showing improved learning in the GBL intervention group.

The findings further indicated high learner satisfaction with the GBL. Among QUIS domains, ‘software learnability’ received the highest scores, likely due to the visual representation of tools and surgical steps, opportunities for active engagement and practice, and embedded feedback within the game. Tavares [15] also concluded that GBL enhances learner satisfaction.

## Strengths and limitations

This study introduces a novel and effective game-based learning framework that addresses a critical gap in surgical nursing education by providing an integrated, cognitively oriented, and procedurally precise training simulation aligned with the complex team dynamics of laparoscopic surgery. The game was deliberately developed as a targeted educational intervention based on clearly defined and scaffolded learning objectives (see Table 1). Its two principal components—“Puzzle of Surgical Nursing Tasks” and “Puzzle of Surgical Instrumentation”—were thoughtfully designed to complement each other, supporting a systematic progression from understanding nursing roles to achieving proficiency in instrument identification and application. This structured approach facilitates learners in building a cohesive and comprehensive mental model of the entire laparoscopic procedure. Future research should evaluate the long-term retention of acquired skills and their direct influence on clinical performance in the operating room.

The limitations of this study include a small sample size and the intervention being conducted at a single institution, which limits the generalizability of the findings. Besides, the assessment focused solely on short-term learning effects and did not evaluate skill retention. The sample size was also limited, which further impacts the generalizability of the results. Furthermore, the online format of the game requires internet access, potentially limiting accessibility for some students.

## Conclusion

This game focuses on teaching the surgical nurse’s tasks in abdominal laparoscopic procedures—including cholecystectomy, appendectomy, inguinal hernia repair, and sleeve gastrectomy—by integrating instrumentation knowledge and application at each surgical stage corresponding to the nurse’s roles. Through puzzle construction mirroring key activities in each surgical phase, learners actively engage with task sequences and instrument functionalities. The results indicate that digital game-based learning significantly improves the reasoning development of surgical nursing students, demonstrating a strong educational effect. It is recommended to implement game-based learning in clinical education curricula focused on laparoscopic surgery.

## Supporting information

S1 Appendix Appendix. (DOCX)
