# Supplementary material for: Effects of digital game-based learning as a tool for laparoscopy training in surgical nursing
Source: PLoS One. 2026 Feb 3;21(2):e0336400. doi: 10.1371/journal.pone.0336400 (PMC12867266; doi:10.1371/journal.pone.0336400)
Supplement: S1 Appendix — (DOCX) [file pone.0336400.s001.docx]

**Appendix 1**

| 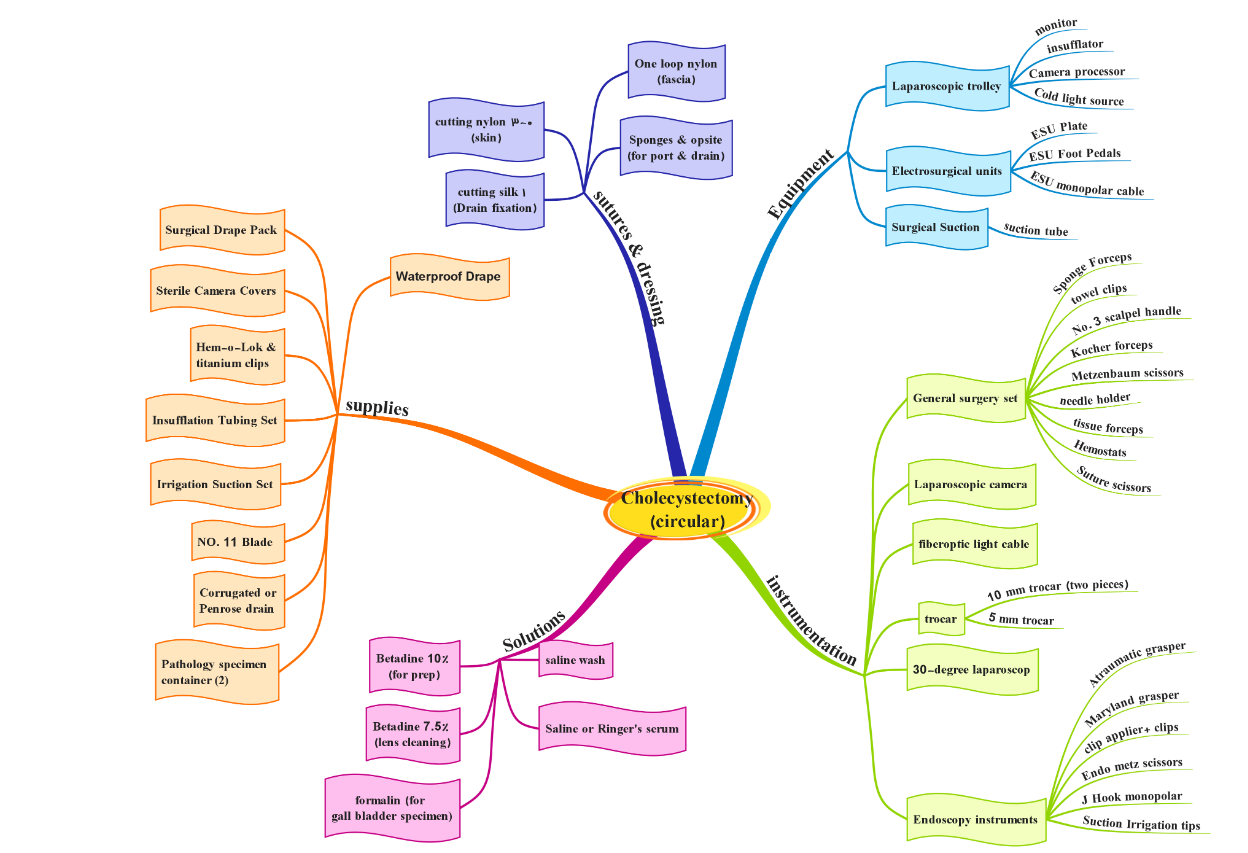 | |
| --- | --- |
| 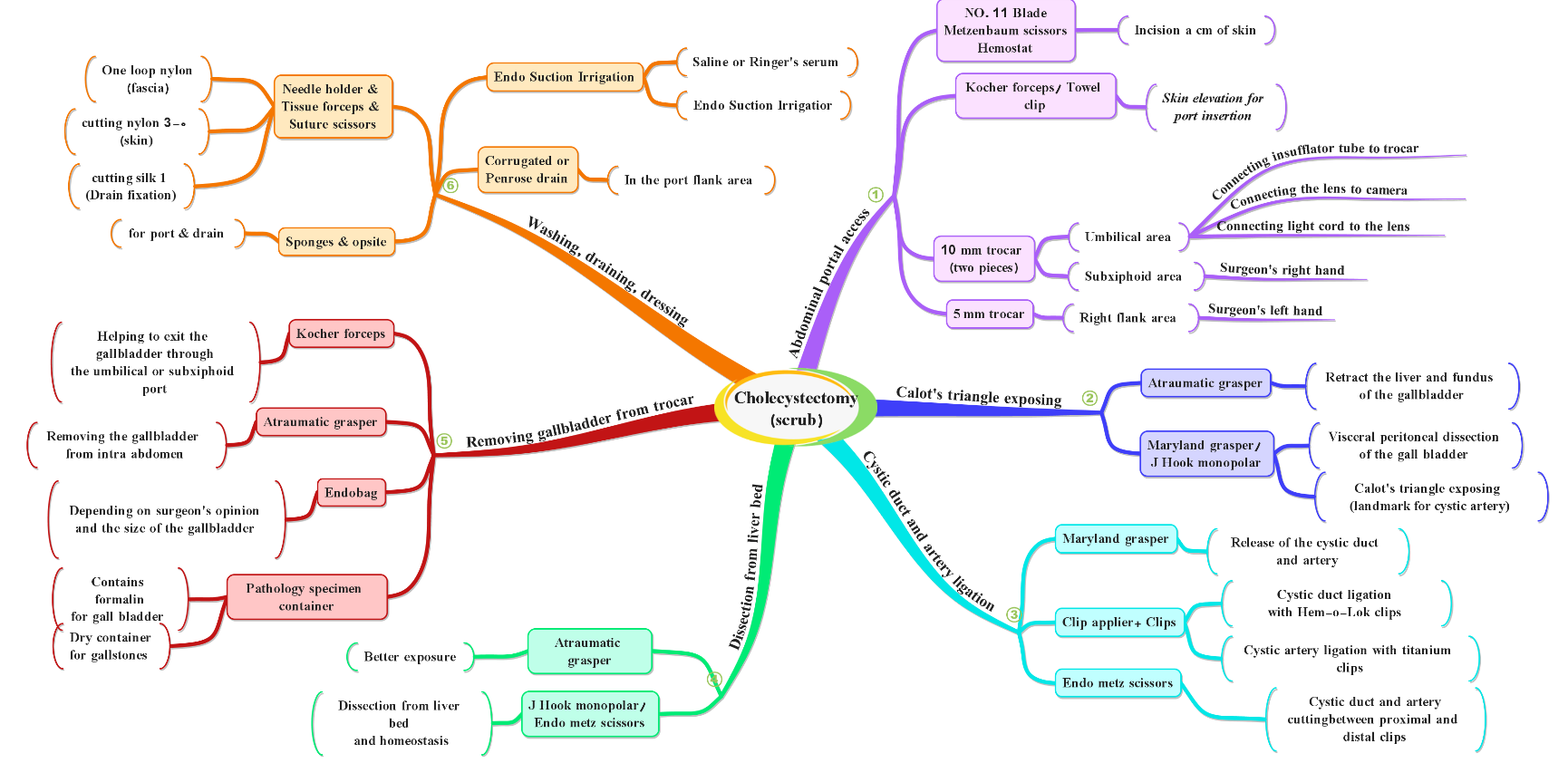 | |
| 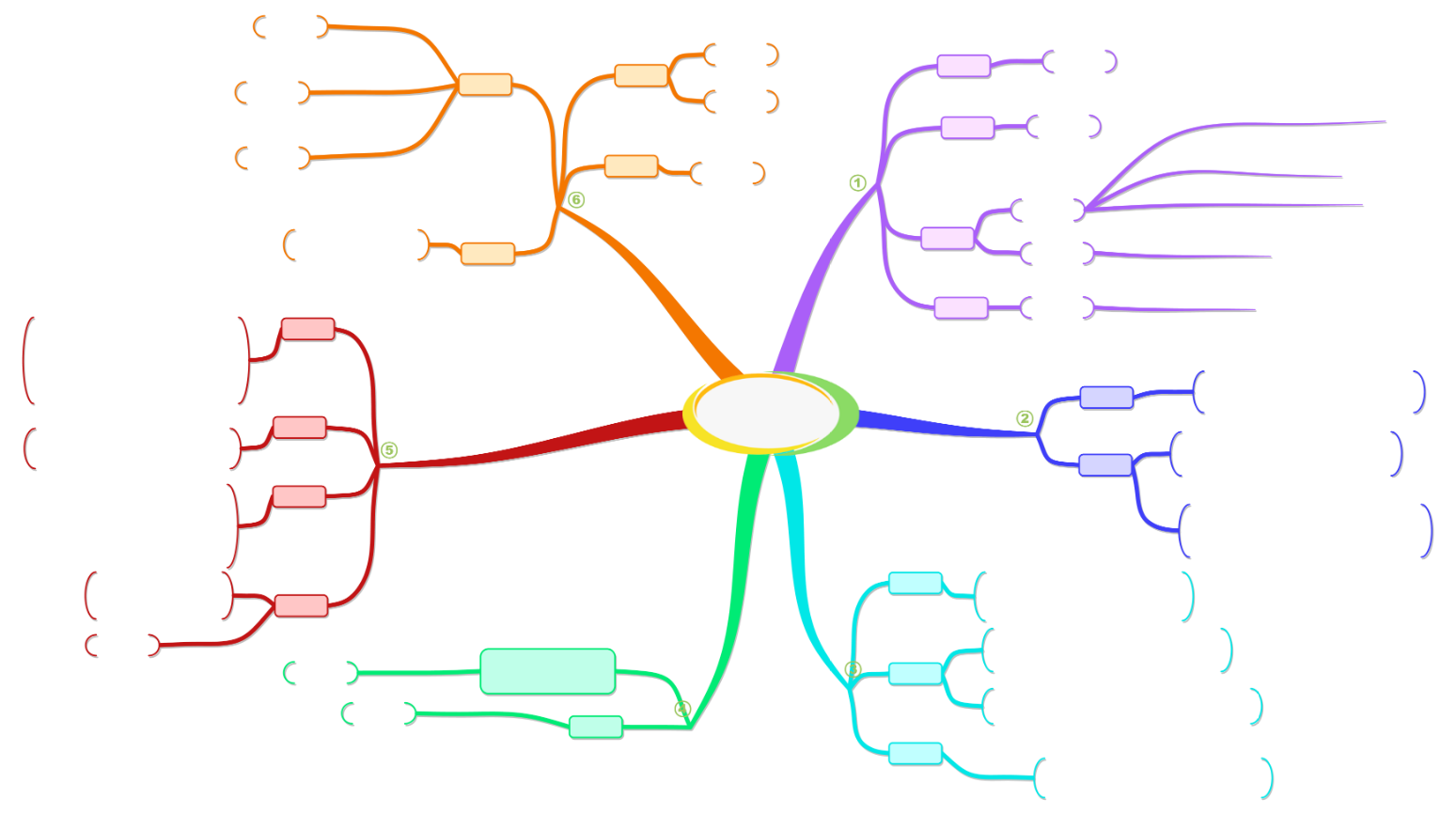 | |
| 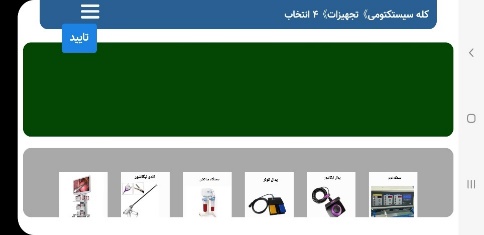 | 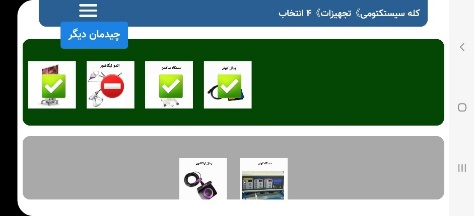 |

Figure 2-

A sample of puzzles in the GBL

**Appendix 2**

This Game is an online system and can be used on smartphones with Android and iOS operating systems. In this way, an installable version (less than 2 MB) was designed for the Android operating system, and a web application was designed for the IOS operating system, accessible at <https://co-surgery.ir/>. The Ubuntu server, Nginx web service, and MySQL database produced the electronic package. The PHP programming language and its Laravel framework were chosen to design a web application capable of interactive features such as different levels of user access, user registration and creation, user panels, data storage, and others. Java programming language was utilized for designing the Android version.

GBL
